# Enterolignans Improve the Expression of Iron-Related Genes in a Cellular Model of Inflammatory Bowel Disease

**DOI:** 10.3390/ijms262211153

**Published:** 2025-11-18

**Authors:** Ottavia Bonuccelli, Anna Gogna, Stefania Mitola, Giulia Abate, Fabiana Ferrari, Francesco Bertagna, Maria Antonia De Francesco, Eugenio Monti, Roberto Bresciani, Giorgio Biasiotto

**Affiliations:** 1 Department of Molecular and Translational Medicine, University of Brescia, 25123 Brescia, Italy; 2 Highly Specialized Laboratory, ASST Spedali Civili di Brescia, 25123 Brescia, Italy; 3 Pediatrics, Mother’s and Baby’s Health Department, Poliambulanza Foundation Hospital Insitute, 25123 Brescia, Italy; 4 Nuclear Medicine, University of Brescia, ASST Spedali Civili, 25121 Brescia, Italy

**Keywords:** inflammatory bowel diseases, anemia, iron metabolism, hepcidin, enterodiol, enterolactone

## Abstract

Anemia is the most common extraintestinal comorbidity of inflammatory bowel diseases (IBDs). Inflammatory cytokines are produced and exert their effects within the bowel microenvironment of IBD patients and, among them, Interleukin 6 (IL-6) induces the expression of hepcidin, encoded by the HAMP gene, contributing to the development of anemia. Enterodiol (END) and Enterolactone (ENL) are enterolignans that are known for their nutraceutical, anti-inflammatory, anti-oxidant, and estrogenic properties. Here, we used the Caco-2 cell line as an in vitro model of bowel disease to evaluate the potential nutraceutical effects of enterolignans on iron metabolism under an inflammatory stimulus induced by IL-6. As is known, IL-6 treatment induces the upregulation of *HAMP* gene expression. Notably, both END and ENL showed a pronounced anti-inflammatory effect, lowering *HAMP* mRNA levels and partially counteracting the effect induced by IL-6. The expression of iron-related genes was also studied to evaluate the effects on iron metabolism. IL-6 downregulated the expression of almost all genes studied. Notably, END and ENL mitigated the effect of IL-6, ameliorating the expression level of *HAMP*, *FTH1*, and *ACO1*, in addition to other END- or ENL-specific effects. The results of this study evidenced the interesting anti-inflammatory properties of enterolignans, which improved the iron homeostasis during inflammation, suggesting a possible role in the management of IBD patients with anemia.

## 1. Introduction

Inflammatory bowel disease (IBD) is a family of inflammatory diseases with a chronic course that can be further classified into Ulcerative Colitis (UC) and Crohn’s Disease (CD). Although the exact cause of the multifactorial etiopathogenesis is still unknown, different environmental factors should also be considered. The primary causes appear to be connected to the immunological and genetic background [1,2]. The clinical management of patients can be often complicated by unpredictable evolution of these chronic disorders. A discontinuous, transmural inflammatory state is visible in all regions of the gastrointestinal tract and is the main histopathological characteristic of CD. Conversely, UC is defined by a more persistent and superficial inflammation of the wall of the colon that primarily affects the mucosal and submucosal layers [3,4]. The prevalence of IBD is expected to increase in the next years, with over two million North Americans and approximately the same number of Europeans currently affected [5].

Anemia is the prevalent comorbidity in IBD, and it has been linked to diminished quality of life and impaired cognitive functions; it imposes a significant burden on these conditions. Anemia in IBD may be the result of a single pathological condition or a combination of factors, including iron deficiency anemia (IDA), anemia of chronic disease (ACD), and anemia associated with the malabsorption or absence of nutritional factors such as folic acid or vitamin B12 [6,7]. The risk of developing anemia may be associated with different age groups, particularly patients between 18 and 25 years and those over 65 years. ACD is more common in CD patients, whereas IDA seems to be more prevalent in UC patients, suggesting that the type of anemia and the type of IBD may be related [8]. There is still uncertainty regarding the prevalence of anemia in IBD, which can range from 36% to 90% [9]. Nevertheless, anemia in IBD may also be the result of iron deficiency or inflammation. Despite the potential for these two forms of anemia to coexist in patients, the clinical challenge of distinguishing between IDA and ACD persists in IBD [10].

Iron metabolism is complex and finely tuned by hepcidin (encoded by the *HAMP* gene), which is its master regulator. According to current theory, this peptide functions like a hormone that controls iron metabolism by sensing the iron level of the body and consequently regulating iron absorption and compartmentalization. The liver primarily produces hepcidin when iron levels and storage increase, and it acts by blocking iron absorption and release by deposits, resulting in decreased iron blood levels. The molecular mechanism involves the binding of hepcidin to ferroportin, the sole known iron exporter from cells, which results in its internalization and destruction. After ferroportin degradation, iron that is present in enterocytes, in hepatocytes, and in macrophages cannot be released into the bloodstream for binding to transferrin, which allows the regulation of the body’s total iron levels. In contrast, the synthesis of hepcidin is inhibited when the body needs iron, such as in iron deficiency, hypoxia, and anemia, or when erythropoietic activity needs to be increased. This allows enterocytes to absorb iron and spleen macrophages and stores to release iron [11]. Genetic iron overload, also known as hereditary hemochromatosis, is the result of mutations in the genes, mainly HFE, that encode the proteins that are involved in the iron-sensing complex acting to control the amount of iron in the bloodstream [12,13]. Inflammation can also influence iron metabolism, and Interleukin 6 (IL-6) has been identified as a significant inducer of hepcidin production through the JAK/STAT3 inflammatory pathway. This innate immunity mechanism reduces the quantity of iron in the bloodstream and the extra-cellular compartment, thereby subtracting it from potential pathogens and resulting in anemia of inflammation. Hepcidin behaves as a type II acute-phase protein in this inflammatory setting [14].

Considering the need to compensate for the losses to avoid deficiencies, the iron absorption is finely tuned. Iron introduced by diet can be of two types: heme or inorganic iron. Heme iron is an important source of this micro-element, and the role of its transporter HCP1 is not fully understood and it is still under debate [15,16]. Inorganic iron is present mainly in vegetable foods, and it is in the oxidized state Fe^3+^, and before the absorption, it must be reduced to Fe^2+^ by cytochrome b reductase 1 (encoded by *CYBRD1*). The Fe^2+^ is then absorbed by ferrous ion membrane transporter 1 (*DMT1*) and enters in the enterocytes. The iron adsorbed can be used for the needs of these cells or can be stored in ferritin (formed by 2 types of subunits codified by *FTL* and *FTH1* genes). Furthermore, it can cross the cytoplasm in the form of a labile iron pool to reach the ferroportin channel (codified by *SLC40A1* gene) in the basolateral membrane where it is released in blood after re-oxidation by hephaestin to bind its transporter, transferrin. Considering that iron is a powerful pro-oxidant, cellular iron homeostasis is finely controlled to readily respond to the needs of the cells. When iron content is high, the cells promote its storage in the ferritin protein, while when its quantity is low the iron uptake is maximized. The control of the optimal iron concentration in the cells, including enterocytes, is handled by the iron-related proteins. The amount of these proteins is controlled by the expression of the relative genes codifying them. Some of the mRNAs of these proteins are controlled at the post-transcriptional level to quickly respond to cellular iron disequilibrium. This control mechanism is mediated by the binding of iron regulatory proteins IRP1 and IRP2 (codified by the *ACO1* and *IREB2* genes, respectively) to a stem-loop structure called the iron regulatory element (IRE) located in the untranslated region (UTR) of the mRNAs. When the iron level is low in the cell, IRPs bind to the IRE structure; when iron is high, IRPs leave the IRE, which releases mRNAs, and consequently IRP1 becomes a cytoplasmic aconitase while IRP2 is degraded. The position of the IRE in the mRNA determines the specific type of control. For example, the mRNAs of ferritin subunits have an IRE structure in the 5′-UTR so, when the iron is low and IRPs are bound to the IRE, the translation is impeded. By contrast, when the iron is high, the IRE is released by IRPs, allowing mRNA translation and ferritin production, and therefore the iron is promptly stored. Conversely, mRNA of the transferrin receptor (codified by the *TFRC* gene) carries the IREs in the 3′-UTR, so when iron is low, IRPs are bound, stabilizing the mRNA and permitting transferrin receptor synthesis; when the iron is high, IRPs are released and the mRNA is degraded (reviewed in [11,17]).

Lignans are a class of diphenolic compounds that are commonly considered to be part of a healthy diet and represent only a small part of the phenolic compounds introduced by foods. They can be found in foods often rich in fiber, such as cereals, nuts, vegetables (brassica), fruits (berries), and beans, or in oils like oilseeds and virgin olive oil [18,19]. These molecules are known to have many interesting bioactivities such as anti-inflammatory, anti-cancer, anti-oxidant, and metabolism-improving effects [20,21]. Enterolignans are represented by Enterodiol (END) and Enterolactone (ENL), and they derive from the metabolization of diet lignans by the gut microbiota mainly in the proximal colon. A part of enterolignans can pass the membrane of the enterocytes and passively diffuses in the biological fluids; another part can be metabolized by glucuronidation or sulfurization, forming soluble conjugates that are actively transported across the basolateral membrane of enterocytes to reach the circulation to be systematically distributed by the portal vein. Enterolignans may be further glucuronidated and sulfureted in hepatocytes and actively transported to bile, activating enterohepatic recirculation. Then, by systematic circulation, they can be adsorbed by various tissues and express their beneficial biological effects [22,23]. Therefore, END and ENL hold great potential to be able to directly act in the bowel or to target all the organs and the tissues exerting in the whole body their nutraceutical properties. In this study, we analyzed the possible role of END and ENL in improving the effects of IL-6-induced inflammation on iron metabolism in a model of IBD.

## 2. Results

### 2.1. Effects of IL-6 Treatment on Iron Metabolism Gene Expression in the Caco-2 In Vitro Model

**Figure 1:**
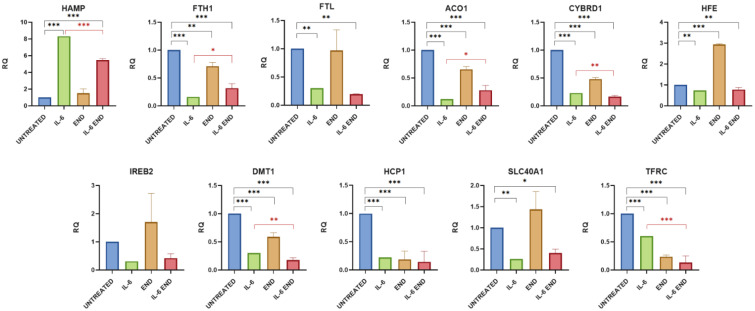
Gene expression analysis of iron metabolism following END treatment. Cells were exposed to inflammatory stimulus and treated with END. The graphs show gene expression levels measured by real-time PCR. Data are presented as relative quantification (RQ), normalized to the housekeeping gene β-tubulin, and expressed relative to untreated controls. Statistical significance is indicated as follows: * *p* < 0.05, ** *p* < 0.01, *** *p* < 0.001. The black color is associated for the comparison of Untreated vs. IL-6+ END and red one for IL-6 vs. IL-6+END.

**Figure 2:**
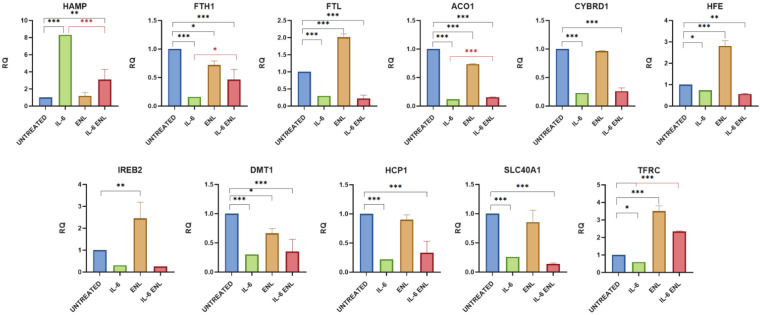
Gene expression analysis of iron metabolism following ENL treatment. Cells were exposed to inflammatory stimulus and treated with ENL. The graphs show gene expression levels measured by real-time PCR. Data are presented as relative quantification (RQ), normalized to the housekeeping gene β-tubulin, and expressed relative to untreated controls. Statistical significance is indicated as follows: * *p* < 0.05, ** *p* < 0.01, *** *p* < 0.001. The black color is associated for the comparison of Untreated vs. IL-6+ENL and red one for IL-6 vs. IL-6+ENL.

Treatment with IL-6 enabled the assessment of the contribution of inflammation mediated by this cytokine in Caco-2 cells, used as an in vitro model of bowel disease. IL-6 had a significant effect on the expression of almost all genes involved in iron metabolism. The expression of the *HAMP* gene codifying hepcidin mRNA increased in a statistically significant manner in all experiments (RQ comparison of END and ENL groups: *p* value ≤ 0.0001) (Figure 1 and Figure 2). The expression of all other genes tested decreased. In particular, the expression of *FTH1*, *FTL*, *ACO1*, *CYBRD1*, *HFE*, *SLC40A1*, *DMT1*, *HCP1 (SLC46A1)*, and *TFRC*, codifying, respectively, for subunits H and L of the ferritin, Aconitase 1 (IRP1, iron regulatory protein 1), cytochrome B1, Homeostatic Iron Regulator (HFE), ferroportin, ferrous ion transport protein 1 (DMT1), heme carrier protein (HCP1), and transferrin receptor, decreased in a statistically significant way. Only *IREB2* codifying for IRP2 protein showed a decreasing trend despite not being statistically significant (Table 1 and Table 2) (Figure 1 and Figure 2).

#### 2.1.1. Effects of Enterodiol on Iron Metabolism Gene Expression in the Caco-2 In Vitro Model

The treatment with END showed an important effect on *FTH1*, *ACO1*, *CYBRD1*, *DMT1*, *HCP1*, and *TFRC* expression, causing it to significantly decrease (*p* value = 0.0011, 0.0004, <0.0001, <0.0001, <0.0001, and <0.0001, respectively), and on the expression of *HFE*, causing it to increase (*p* value < 0.0001). The expression of *SLC40A1* tended to increase without reaching statistical significance (*p* value = 0.0907). When cells were treated with IL-6 and END together for 48 h, it reduced or reversed the pro-inflammatory effect of this cytokine, leading to an improvement or normalization of the expression of the analyzed genes. The mRNA level of hepcidin (*HAMP* expression) decreased in a statistically significant manner (*p* value < 0.0001), without returning to the normal level, and the difference with the physiological expression remained significant (*p* value < 0.0001). *FTH1* and *ACO1* increased their expression (*p* value = 0.0174 and 0.0149, respectively) without returning to the normal level (both *p* values < 0.0001). *CYBRD1*, *DMT1*, and *TFRC* showed a further decrease in their expressions, likely due to the sum of the effects of IL-6 and END (*p* value = 0.0053, 0.0081, and <0.0001, respectively). The treatment with END did not change the effect of IL-6 inflammation on the expression of *FTL*, *HFE*, *IREB2*, *HCP1*, and *SLC40A1*. (Table 1 and Figure 1).

#### 2.1.2. Effects of Enterolactone on Iron Metabolism Gene Expression in the Caco-2 In Vitro Model

The addition of ENL to the cell culture medium showed a similar effect to that observed for END. The expression of the *FTH1*, *ACO1*, and *DMT1* genes decreased (*p* value = 0.0189, <0.0001, and 0.0171, respectively), and the mRNA level of *HFE* and *TFRC* increased (*p* value for both genes <0.0001) also using this enterolignan. When ENL was added together with IL-6, *HAMP* expression showed a significant decrease (*p* value < 0.0001), although it remained higher than in non-treated cells (*p* value = 0.0063). The expression of the other genes analyzed fluctuated, and only *FTH1*, *ACO1*, and *TFRC* showed an increase in expression that was statistically significant (*p* value = 0.0128, 0.0002, and <0.0001, respectively), but without reaching the physiological expression for *FTH1* and *ACO1* (*p* value = 0.0007 and <0.0001, respectively). The amount of *TFRC* mRNA increased instead from the normal level in a significant way (*p* value < 0.0001) (Table 2 and Figure 2).

## 3. Discussion

Anemia significantly impacts the clinical status of patients with inflammatory bowel disease (IBD) and represents one of the most common comorbidities associated with these conditions, as well as with other chronic inflammatory disorders. Patients have a heavy burden from this condition, which results in a poor quality of daily life, mood disorders, general weakness, and impaired cognitive functions. Anemia in IBD is brought on by several conditions that frequently coexist, including chronic intestinal bleeding, impaired absorption of vitamin B12 and/or folate, and inflammation of the gastrointestinal mucosa. Iron deficiency is another significant condition that accounts for at least half of anemia cases, which may support the use of iron supplements in the treatment of these patients. Moreover, anemia is associated with a more severe form of IBD, various complications, and worse prognosis [24].

Iron therapy is suggested by IBD clinical guidelines to correct hemoglobin levels and red blood cell parameters. However, due to the non-negligible role of IL-6-mediated molecular mechanisms, oral iron is not fully absorbed. The unabsorbed fraction remains in the intestinal lumen, where it can cause mucosal damage and disrupt the microbiota balance. Moreover, increased levels of pro-inflammatory cytokines were observed in animal models of IBD. To bypass the intestinal problem of absorption and to maximize the patient support, the intravenous administration of iron formulation is recommended in patients with severe anemia with hemoglobin values below 10 g/dL [1,6,25].

An important role of inflammation in IBD and other bowel diseases is determined by infiltration of macrophages, mesenchymal cells, and T lymphocytes and by the contribution of the intestinal epithelial cells of the mucosa, which produce IL-6 and other inflammatory cytokines. IL-6 effects were extensively studied in Caco-2 cells and in IBD patients [26,27]. IL-6 inflammation has two main effects on iron metabolism that can be connected with anemia: (a) the systemic effect caused by the liver’s production of hepcidin that determines compartmentalization of iron in macrophages and stores, impeding iron absorption by the bowel and lowering blood iron levels; (b) the local effect, often resulting from local inflammation, that is mediated by hepcidin production within the inflamed tissues through autocrine and paracrine mechanisms [28,29].

Considering the aspects discussed above, we employed the Caco-2 cell line as an in vitro model of the human intestinal epithelium to investigate the effects of IL-6-mediated inflammation, with a particular focus on its impact on iron metabolism. In fact, these observations may be very important to hypothesize the specific effect of local inflammation on iron absorption as a specific aspect of a more complex process influenced by bleeding, malabsorption, and systemic regulation. As expected, the treatment with IL-6 significantly increased the expression of hepcidin, the master regulator of iron metabolism [30,31,32]. The pro-inflammatory effect lowered the expression of all the genes studied and in almost every one in a statistically significant way (*FTH1*, *FTL*, *ACO1*, *CYBRD1*, *HFE*, *DMT1*, *HCP1*, *SLC40A1*, and *TFRC*). These decreases underlined a great effect of IL-6 on Caco-2 cellular iron metabolism.

The increase in hepcidin production of Caco-2 cells under IL-6 stimulus could free hepcidin in the small volume of the cell culture medium of the wells, reaching a sufficient concentration to activate autocrine and paracrine effects, determining ferroportin degradation and consequent cellular iron retention. This mechanism could be very important because it could mimic what probably happens in vivo, where the inflammation mediated by IL-6 could increase local hepcidin, activating the autocrine and paracrine effect, which may lower iron absorption by degradation of ferroportin and may contribute to worsening the anemia grade. The consequent increase in intracellular iron could likely explain the diminutions of the expression of some iron-related genes, such as *DMT1*, *HCP1*, *SLAC40A1*, and *TFRC*. The potential causes of the decrease in expression of the genes under investigation may vary. The expression of ferritin is not influenced by IL-6, but its translation is induced by a specific sequence in the mRNA [33]. Nevertheless, we could hypothesize that, during the time of treatment, the increased amount of iron in the cells involved the displacement of IRPs from the IRE sequence of their mRNAs, activating the translation and increasing the ferritin synthesis until the necessary amount was obtained to store the excess of cytoplasmic iron. The literature reports that inflammation and hepcidin can induce ferroptosis in colonocytes, in animal models, and in Caco-2 cells, increasing the labile iron pool and lipid peroxidation [34]. In addition, inflammation may increase the intracellular iron, activating the process of ferroptosis. In this condition, ferritin protein increases while FTH1 mRNA decreases as well as SLC40A1 mRNA in Caco-2 cells [35]. Moreover, intracellular iron may stimulate mRNA turnover mechanisms, resulting in enhanced degradation or specific transcripts. In addition, the increase in the labile iron pool could reduce the stability of mRNA by the degradation induced by reactive oxygen species (ROS) [36,37,38]. It is reported in the literature that hepcidin administered on duodenal biopsies induces a decrease in the expression of *CYBRD1* (DCYTB), *DMT1*, *HCP1*, *SLC40A1* (FPN1), and other iron-related genes [16]. In addition, we cannot exclude a larger effect of these variables in decreasing the expression of the other iron-related genes, such as *ACO1* [39,40,41]. Another important factor in this study was the fact that the cells were treated at confluence for a relatively long time (48 h), and this condition may contribute to the modulation of the expression of at least *FTH1*, *FTL*, and *TFRC* if compared with cells in active proliferation, such as cancer cells [42,43].

Some nutraceutical molecules contained in functional foods are studied for their properties to ameliorate bowel inflammation and iron absorption, relieving anemia burden [32,44]. Interestingly, the enterolignans ENL and END belong to this family of molecules, and they had great effects on the expression of iron-related genes and then on iron metabolism in our model. In fact, END reduced the effect of inflammation induced by IL-6 on *HAMP* expression and then lowered the hepcidin production, improving the expression without restoring the physiological condition and confirming its anti-inflammatory effect. Moreover, the expression of some iron-related genes significantly increased, such as *FTH1* and *ACO1*. In addition, we could notice a further significant decrease in *CYBRD1*, *DMT1*, and *TFRC*.

A similar effect on *HAMP* expression was also observed upon administration of ENL, which has been confirmed as a highly interesting anti-inflammatory molecule. The ENL effects were slightly different from END; in fact, *FTH1*, *ACO1*, and *TFRC* increased their expression without returning to the basal level.

It is known that enterolignans can act as phytoestrogens, binding both the estrogen receptors alpha and beta [45], and they can act as phytoestrogens. Estrogens have an important role in regulating the expression of some iron-related proteins. *HAMP* promoter has two estrogen regulatory elements (EREs), the first between −2474 and −2462 bases and the second between −144 and 1232 bases, and estrogens decrease the expression of hepcidin [46,47]. Estrogens have a fundamental biological role in controlling iron metabolism. In fact, they lower hepcidin production, permitting the maximization of the iron absorption, and then help women of childbearing potential to counteract the iron losses caused by menstruation and preserve their iron stores. This effect of inhibiting hepcidin expression was also observed for 7-hydroxymatairesinol in the Caco-2 cell line [32]. Moreover, *SLC40A1* also has a functional ERE located in its promoter, and estrogen decreased the expression of ferroportin in the TPH1 cell line (human leukemia monocytic cell) and in macrophages [48]. Therefore, we could hypothesize a contributing role of the estrogenic effect of enterolignans in regulating the expression of the *HAMP* and *SLC40A1* genes. The anti-inflammatory effect of enterolignans may be partially mediated by their estrogenic effect. In fact, 17-β-estradiol, via estrogen receptors, decreases the Toll-like receptor 4 signaling pathway, inhibiting pro-inflammatory cytokines including IL-6 [49], and enterolignans could hypothetically activate a similar anti-inflammatory mechanism.

All this evidence showed the capacity of the enterolignans, both END and ENL, to decrease inflammation induced by IL-6, improving iron metabolism in Caco-2 cells used as a model of bowel disease. As reported above, the increase in local hepcidin production in an inflamed bowel by IL-6 effects could activate an autocrine and paracrine mechanism that may cause ferroportin degradation. This local hepcidin may act in addition to that released by systemic control of the liver. Consequently, the release of iron is inhibited, which may exacerbate systemic iron deficiency and contribute to the worsening of anemia. The anti-inflammatory effects of both END and ENL may improve iron absorption by reducing local hepcidin levels in the intestine, contributing to relieving the anemia burden. Additionally, given their ability to circulate in the bloodstream and reach the liver via the portal vein, these compounds may also have systemic effects. The anti-inflammatory effect of lignans is known, and 7-hydroxymatairesinol was studied in the past by our group for its effect on general metabolism in animal models and for its specific effect on *HAMP* expression in the Caco-2 cell line [32,50]. All the lignans introduced by diet, therefore 7-hydroxymatairesinol, are metabolized by microbiota into enterolignans, namely, END and ENL [23,50,51]. This evidence makes the results of this study particularly relevant because it showed the direct effects of enterolignans on iron metabolism in the Caco-2 cells as a model of the intestine. Interestingly, the bowel is the district where enterolignans are produced, and these molecules have the potential to be absorbed and to express their beneficial effects in enterocytes and to pass into circulation, with the potential to target all organs and tissues of the body.

Our work shows some limitations. The first limitation is that we did not verify the effects of changes in gene expressions by analyzing the amount of the relative proteins by Western blotting and/or using other suitable methodologies such as ELISA, flow cytometry, and microscopy, and this in-depth evaluation is planned for the future. Despite the production of some of these proteins being regulated at the post-transcriptional level, there is evidence in the literature showing that the expression of the genes, and then the amount of mRNAs, often more than translation, is involved in determining the proteins’ abundance in mammals [16,52,53,54]. Second, the results obtained in the Caco-2 cellular model should also be confirmed in intestine biopsies and in IBD patients to obtain information in a more physiological model of the enterolignans’ beneficial effects. Third, although the panel of the genes studied is sufficiently large, some genes involved in ferroptosis could be added to verify this important pathway [55,56]. Fourth, to complete our study, it will be interesting to measure the LIP and the iron export and retention by measuring the iron concentration in the basolateral medium. Finally, the study of the expression of some genes in this model, such as *HFE*, *ACO1*, and *TFRC*, should be the object of in-depth and dedicated future studies.

## 4. Materials and Methods

### 4.1. Cell Culture and Treatments

The Caco-2 cell line was purchased from the Istituto Zooprofilattico Sperimentale della Lombardia e dell’Emilia Romagna ‘Bruno Ubertini’, Brescia, Italy. Caco-2 cells were maintained in Dulbecco’s Modified Eagle’s Medium (DMEM; Gibco, Thermo Fisher Scientific, Waltham, MA, USA) supplemented with 10% fetal bovine serum (FBS; Gibco, Thermo Fisher Scientific, Waltham, MA, USA), L-glutamine (VWR International, Radnor, PA, USA), and Antibiotic-Antimycotic containing 10,000 units/mL of penicillin, 10,000 μg/mL of streptomycin, and 25 μg/mL of amphotericin B (Gibco, Thermo Fisher Scientific, Waltham, MA, USA). The cultures were incubated at 37 °C in a humidified atmosphere with 5% CO2 and tested with a LookOut Mycoplasma PCR Detection Kit (Merck Life Science, Darmstadt, Germany).

A total of 15,000 cells were seeded in 12-well plates (Sarstedt, Nümbrecht, Germany) for the following treatments. Confluent Caco-2 cells were treated with IL-6 50 ng/mL (Thermo Fisher Scientific, Waltham, MA, USA) [32,57,58] for 48 h to induce an in vitro model of bowel disease. Enterodiol 1 μM (Merck Life Science, Darmstadt, Germany) and Enterolactone 1 μM (Merck Life Science, Darmstadt, Germany) were administered, either in the presence or absence of IL-6 (50 ng/mL) for 48 h to test their biological effects, as previously described for similar molecules in [20,32,50]. All treatments were performed in biological triplicate, for a total of 18 treated samples and 6 untreated controls (Supplementary materials: file 1 Photos of cells 20X -MTT-Table S1 RQ mean + SD).

### 4.2. RNA Extraction and RT-qPCR

After 48 h of treatment, total RNA was extracted using a PureLink RNA Mini Kit (Thermo Fisher Scientific, Waltham, MA, USA), treated with RQ1 Rnase-Free Dnase 1000 u (Promega, Milan, Italy) and reverse-transcribed into cDNA using M-MLV Reverse Transcriptase 10,000 u (Thermo Fisher Scientific, Waltham, MA, USA). Quantitative real-time PCR (RT-PCR) was performed to analyze mRNA expression levels using iTaq™ Universal SYBR^®^ Green Supermix (Bio-Rad, Hercules, CA, USA). The retrotranscribed RNA amount was 1 µg. The cDNA for real-time PCR was diluted to obtain a final concentration of 0.005 µg/µL. The protocol for technical triplicates involves the following reaction mixture:

- 4 µL of cDNA;
- 6 µL of master mix, consisting of the following:
  ○ 16.25 µL SYBR Green PCR mix;
  ○ 0.325 µL of 10 µM concentrated primers;
  ○ 2.925 µL of nuclease-free water.

The primer sequences employed for real-time PCR are presented in Table 3. Primer pairs were previously assessed by conventional PCR. The instrument was a ViiA 7 Real-Time PCR System, and the abnormalities software was Design & Analysis 2 (DA2) software. The protocol applied for real-time PCR was as follows: Hold Stage: 50.0 °C for 02:00 min and 95.0 °C for 10:00 min, followed by 40 cycles of PCR Stage: 95.0 °C for 00:15 s with 60.0 °C for 01:00 min and finish with Melt Curve Stage: 95.0 °C for 00:15 s, 60.0 °C for 01:00 min and 95.0 °C for 00:15 s. The data were normalized using the housekeeping gene β-tubulin and fold change values (relative quantification, RQ) were determined in comparison to the untreated cells (for untreated and IL-6 the CT of the 18 biological replicates was grouped and used for the calculation of RQ). The real-time PCR protocol was optimized by the institute. This qPCR experiment was conducted in compliance with the MIQE guidelines as closely as possible. (Supplementary materials: file 1 Photos of cells 20X -MTT-Table S1 RQ mean + SD).

### 4.3. Statistical Analyses

Statistical analyses were performed using GraphPad Prism 8 software (Dotmatics, MA, USA). One-way ANOVA with Dunnett’s multiple comparison test analysis was used to evaluate treatment differences, using data obtained from experiments performed in biological triplicate. Statistical significance was set at *p* < 0.05.

## 5. Conclusions

In conclusion, the data obtained from this study presented an interesting description of the effects of IL-6-induced inflammation on iron metabolism in an in vitro model of IBDs, highlighting the significant anti-inflammatory properties of END and ENL. In vitro experiments confirmed that END and ENL can ameliorate the imbalance of iron-related genes such as FTH1 and ACO1 by inhibiting the IL-6-mediated JAK/STAT3-HAMP pathway, thereby regulating local intestinal iron homeostasis under inflammatory conditions, contributing to relieving anemia burden. These enterolignans improve the homeostasis of iron metabolism during inflammation, suggesting a potential role in the management of IBD patients with anemia.

## Figures and Tables

**Table 1:** *p* values of the relative quantification (RQ) of the expression of genes in Caco-2 cells treated with IL-6 and END.

Genes	Untreated vs. IL-6	Untreated vs. END	Untreated vs. IL-6+END	IL-6 vs. IL-6+END
*HAMP*	**<0.0001**	0.1525	**<0.0001**	**<0.0001**
*FTH1*	**<0.0001**	**0.0011**	**<0.0001**	**0.0174**
*FTL*	**0.0081**	0.9951	**0.0070**	0.8874
*ACO1*	**<0.0001**	**0.0004**	**<0.0001**	**0.0149**
*CYBRD1*	**<0.0001**	**<0.0001**	**<0.0001**	**0.0053**
*HFE*	**0.0015**	**<0.0001**	**0.0043**	0.6591
*IREB2*	0.3025	0.2930	0.4234	0.9870
*DMT1*	**<0.0001**	**<0.0001**	**<0.0001**	**0.0081**
*HCP1*	**0.0001**	**<0.0001**	**<0.0001**	0.7772
*SLC40A1*	**0.0077**	0.0907	**0.0238**	0.7600
*TFRC*	**0.0001**	**<0.0001**	**<0.0001**	**<0.0001**

The *p* values reaching statistical significance are reported in bold. The *p* values that tended to be statistically significant are underlined.

**Table 2:** *p* values of the relative quantification (RQ) of the expression of genes in Caco-2 cells treated with IL-6 and ENL.

Genes	Untreated vs. IL-6	Untreated vs. ENL	Untreated vs. IL-6+ENL	IL-6 vs. IL-6+ENL
*HAMP*	**<0.0001**	0.9308	**0.0063**	**<0.0001**
*FTH1*	**<0.0001**	**0.0189**	**0.0007**	**0.0128**
*FTL*	**<0.0001**	**<0.0001**	**<0.0001**	0.3677
*ACO1*	**<0.0001**	**<0.0001**	**<0.0001**	**0.0002**
*CYBRD1*	**<0.0001**	0.3262	**<0.0001**	0.4312
*HFE*	**0.0450**	**<0.0001**	**0.0076**	0.2284
*IREB2*	0.1607	**0.0071**	0.1744	0.9966
*DMT1*	**0.0002**	**0.0171**	**0.0003**	0.9074
*HCP1*	**<0.0001**	0.6534	**0.0002**	0.5395
*SLC40A1*	**0.0001**	0.2402	**<0.0001**	0.3596
*TFRC*	**0.0213**	**<0.0001**	**<0.0001**	**<0.0001**

The *p* values reaching statistical significance are reported in bold.

**Table 3:** Primer sequences for genes involved in iron metabolism used in qPCR.

Gene	Primer Forward	Primer Reverse
*FTH1*	5′-AGCTCTACGCCTCCTACGTT-3′	5′-GTGGCCAGTTTGTGCAGTTC-3′
*ACO1*	5′-CAACCCATTCGCACACCTTG-3′	5′-CGAGCAGGCTTAAATGGCAC-3′
*CYBRD1*	5′-GGAGTATGGGGCGCTGATG-3′	5′-TCTGTAGACGATGATGGCGATG-3′
*HFE*	5′-CGCTTCTCCTCCTGATGCTT-3′	5′-AGACCAAGGTCCTGCTCTGA-3′
*IREB2*	5′-TGTTGGAAGCTGCTGTACGA-3′	5′-CGGACAAGCAGGATGGACTT-3′
*HAMP*	5′-TCAAGACCCAGCAGTGGGA-3′	5′-CTCCTTCGCCTCTGGAACAT-3′
*SLC40A1*	5′-CGACTACCTGACCTCTGCAAAA-3′	5′-ACATTCTGTACCACCAGCGA-3′
*FTL*	5′-CCAGCACCGTTTTTGTGGTT-3′	5′-GCCAATTCGCGGAAGAAGTG-3′
*TFRC*	5′-AGAACTACACCGACCCTCGT-3′	5′-TGCCACACAGAAGAACCTGC-3′
DMT1 *	5′-TGCATTCTGCCTTAGTCAAGTC-3′	5′-ACAAAGAGTGCAATGCAGGA-3′
HCP1 *	5′-CCGACCTCGGCTACAATG-3′	5′-CCAGTGGGAGGTAAGGGTCT-3′
*β TUBULIN*	5′-AGATCGGGGCCAAGTTCTGG-3′	5′-CTCGAGGCACGTACTTGTGA-3′

* Primer pairs of DMT1 and HCP1 were referred to in [16].

## Data Availability

The data presented in this study are available on request to the corresponding author (the data are not publicly available due to the policy of our laboratory).

## Supplementary Materials

The following supporting information can be downloaded at: https://www.mdpi.com/article/10.3390/ijms262211153/s1.

## Abbreviations

ACD	Anemia of chronic disease
CD	Crohn’s Disease
IBD	Inflammatory bowel disease
IDA	Iron deficiency anemia
IL-6	Interleukin 6
IRE	Iron regulatory element
END	Enterodiol
ENL	Enterolactone
ERE	Estrogen regulatory elements
ROS	Reactive oxygen species
RQ	Relative quantification
UC	Ulcerative Colitis
UTR	Untranslated region
