# Supplementary material for: Enterolignans Improve the Expression of Iron-Related Genes in a Cellular Model of Inflammatory Bowel Disease
Source: Int J Mol Sci. 2025 Nov 18;26(22):11153. doi: 10.3390/ijms262211153 (PMC12652460; doi:10.3390/ijms262211153)
Supplement: Supplementary file 1 [file ijms-26-11153-s001.zip › ijms-3933219-supplementary.pdf]

END T 48h (magnification 20X)

Untreated T 48h

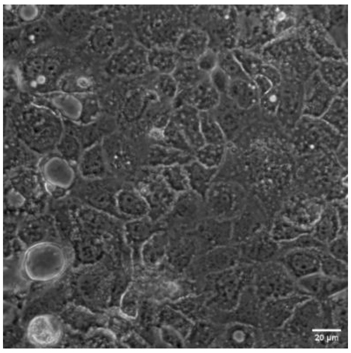

IL-6 T 48h

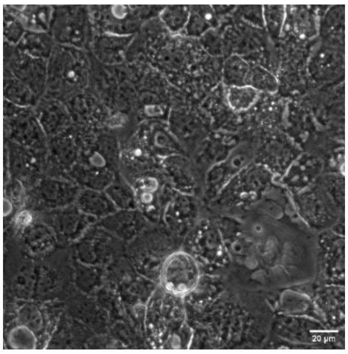

IL6 + END T 48h

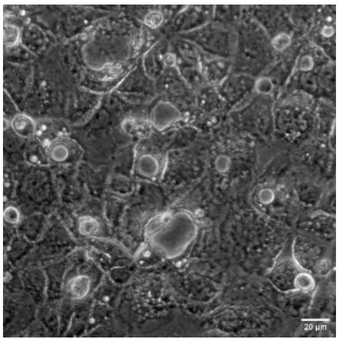

END T 48h

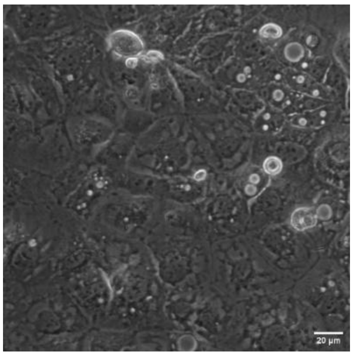

END T 0h (magnification 20X)

Untreated T 0h

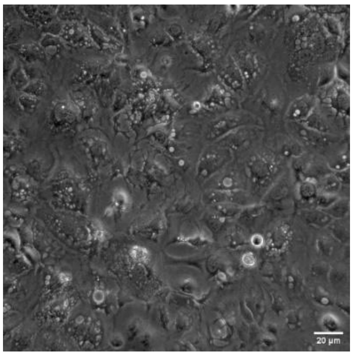

IL-6 T 0h

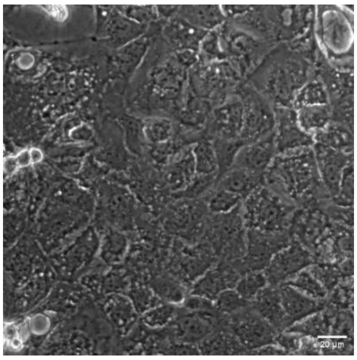

IL6 + END T 0h

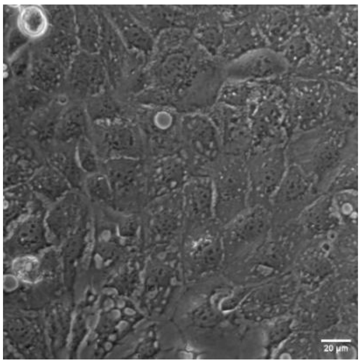

END T 0h

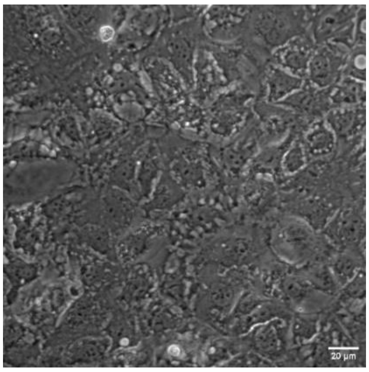

ENL T 48h (magnification 20X)

Untreated T 48h

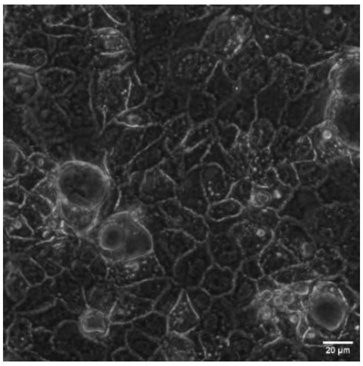

IL-6 T 48h

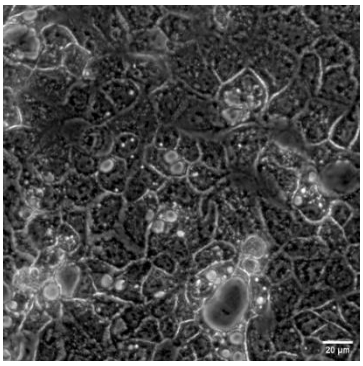

IL-6 + ENL T 48h

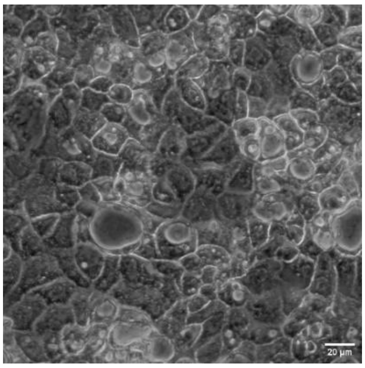

ENL T 48h

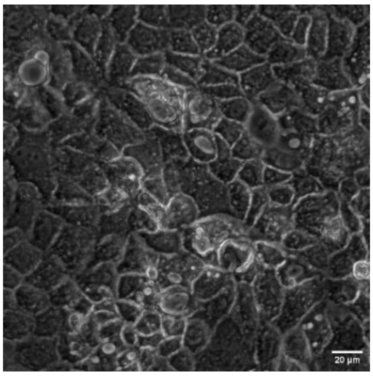

ENL T 0h (magnification 20X)

Untreated T 0h

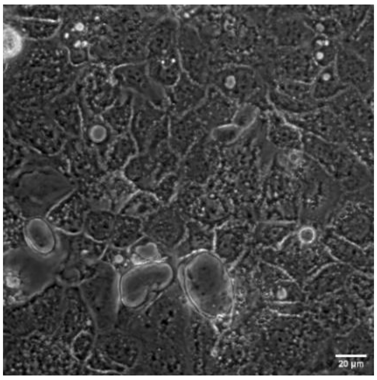

IL-6 T 0h

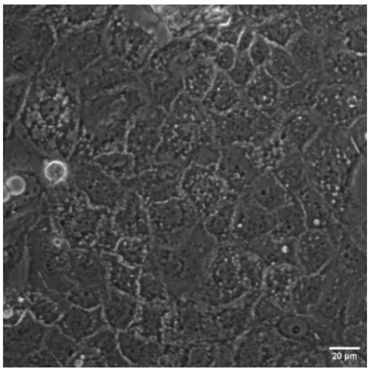

IL-6 + ENL T 0h

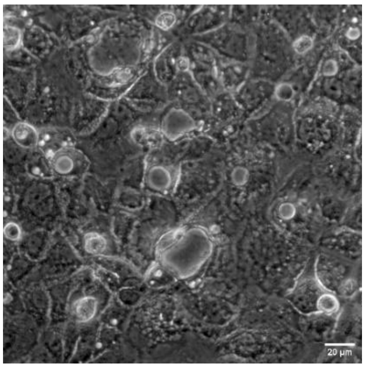

ENL T 0h

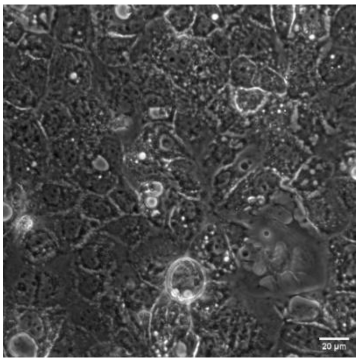

MTT viability assay

Caco-2 cells were seeded at a density of 15,000 cells/cm² in 96-well plates (EuroClone, Italy). After 24 h, cells were treated in triplicate with END, ENT, and IL-6, except for the control, which received DMEM only. Doxorubicin was used as a positive control for cytotoxicity. After 48 h, 100 µL of the 0.5 mg/mL MTT solution (M2128-1G, Sigma-Aldrich, Germany) was added and incubated for 4 h at 37 °C. The solution was then removed, and formazan crystals were dissolved in 100 µL of DMSO (D2650, Sigma-Aldrich, Germany). Absorbance was measured at 570 nm using an Ensight MultiMode Reader (PerkinElmer, Connecticut, USA).

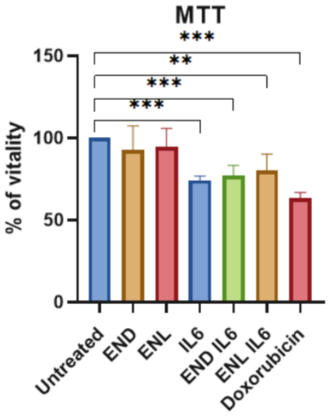

MTT cell viability assay of the treatments. The viability of Caco-2 cells after exposure to IL-6, END, and ENL demonstrates that both enterolignans exhibit no cytotoxic effects when administered individually. Doxorubicin was included as a positive control.

Supplementary Table S1 RQ mean ± SD

Genes	CT UNTREATED ± SD*	CT IL6 ± SD*	RQ END ± SD	RQ IL6+END ± SD	RQ ENL ± SD	RQ IL6+ENL ± SD
HAMP	34,223 ± 2,505	30,018 ± 1,812	1,507 ± 0,517	5,46 ± 0,184	1,197 ± 0,395	3,09 ± 1,188
FTH1	17,742 ± 0,902	19,214 ± 4,434	0,71 ± 0,071	0,317 ± 0,081	0,72 ± 0,071	0,465 ± 0,177
FTL	20,445 ± 3,290	21,3482 ± 3,538	0,97 ± 0,365	0,195 ± 0,007	2,015 ± 0,092	0,225 ± 0,092
ACO1	22,153 ± 0,787	24,044 ± 4,003	0,655 ± 0,049	0,28 ± 0,087	0,735 ± 0,007	0,155 ± 0,007
CYBRD1	29,855 ± 0,740	30,840 ± 3,351	0,48 ± 0,028	0,163 ± 0,025	0,965 ± 0,007	0,26 ± 0,057
HFE	27,525 ± 2,647	26,805 ± 1,361	2,935 ± 0,035	0,783 ± 0,097	2,81 ± 0,240	0,565 ± 0,035
IREB2	23,725 ± 2,132	24,251 ± 3,163	1,7 ± 1,019	0,42 ± 0,159	2,453 ± 0,739	0,25 ± 0,0
DMT1	23,186 ± 1,588	23,486 ± 2,415	0,59 ± 0,071	0,177 ± 0,040	0,663 ± 0,081	0,35 ± 0,213
HCP1	24,134 ± 2,374	24,911 ± 3,670	0,187 ± 0,148	0,143 ± 0,188	0,903 ± 0,081	0,333 ± 0,196
SLC40A1	24,469 ± 3,205	25,580 ± 3,593	1,437 ± 0,422	0,403 ± 0,091	0,855 ± 0,205	0,14 ± 0,014
TFRC	31,701 ± 4,213	31,606 ± 4,926	0,237 ± 0,029	0,133 ± 0,117	3,5 ± 0,311	2,34 ± 0,028

* CT of untreated and IL-6 are respectively unified (18 replicates for each respectively CT± SD); RQ are calculated with the average of the CT and then in Figure 1 and 2 there are no SD. The RQs are calculated with n = 3 biological replicates.
